# The Effect of Developmental Trauma on Brain Structures Involved in Threat and Memory Processing and Its Relation to Psychotic Experiences in Adulthood

**DOI:** 10.1093/schbul/sbaf184

**Published:** 2025-10-28

**Authors:** Ava J C Mason, Paul G Y Jung, Kate Merritt, Arjun Sethi, Stanley Zammit, Derek K Jones, Anthony S David, Michael A P Bloomfield

**Affiliations:** Division of Psychiatry, UCL Institute of Mental Health, University College London, London W1T 7NF, United Kingdom; Division of Psychiatry, UCL Institute of Mental Health, University College London, London W1T 7NF, United Kingdom; Division of Psychiatry, UCL Institute of Mental Health, University College London, London W1T 7NF, United Kingdom; Department of Forensic and Neurodevelopmental Sciences (FANS), Institute of Psychiatry, Psychology and Neuroscience, King’s College London, Denmark Hill, SE5 8AB, United Kingdom; Division of Psychological Medicine and Clinical Neurosciences, Cardiff University, School of Medicine, UHW Main Building, Heath Park, Cardiff, CF14 4XN, United Kingdom; Population Health Sciences, Bristol Medical School, University of Bristol, Bristol, BS8 2PS, United Kingdom; Brain Research Imaging Centre (CUBRIC), Cardiff University, Cardiff, CF24 4HQ, United Kingdom; Division of Psychiatry, UCL Institute of Mental Health, University College London, London W1T 7NF, United Kingdom; Division of Psychiatry, UCL Institute of Mental Health, University College London, London W1T 7NF, United Kingdom; The Traumatic Stress Clinic, St Pancras Hospital, Camden & Islington NHS Foundation Trust, London NW1 0PE, United Kingdom

**Keywords:** developmental trauma, psychotic experiences, sMRI, threat processing, memory processing

## Abstract

**Background and Hypothesis:**

Despite growing evidence of a causal association between developmental trauma (DT) and psychotic experiences (PEs), the precise neurobiological mechanisms underlying this association remain poorly understood. We examined the effect of DT on the structure of brain regions involved in threat and memory processing, and the role of these alterations in the association between DT and PEs.

**Study Design:**

This study used data from the Avon Longitudinal Study of Parents and Children, a large, population-based birth cohort in the United Kingdom. Data were available from 419 participants, including DT reported by the parents or the participants between ages 0 and 17 years, PEs at age 18, and volumes of regions involved in threat and memory processing in adulthood (mean = 21,2, SD = 1.5 years).

**Study Results:**

DT exposure was associated with increased odds of PEs (odds ratio [OR] = 1.64; 95% CI, 1.04-2.59, *P* = .035), with evidence supporting cumulative risk effects for exposure to multiple trauma types (*B* = 0.160, *P* < .001). DT was also associated with reduced left amygdala volumes (*B* = −0.011, *P* = .02) with evidence again supporting cumulative risk effect with multiple trauma types (*B* = −0.006, *P* = .01). Reduced bilateral amygdala volume was associated with an increased odds of PEs driven by the left amygdala (OR = 0.001, 95% CI, 0.000-0.154, *P* = .006).

**Conclusions:**

These findings are consistent with theories that alterations in brain regions involved in threat and memory processing lie on the neurobiological pathway from DT to PEs, offering the possibility of prevention strategies for psychosis.

## Introduction

There is growing evidence that developmental trauma (DT)—psychologically traumatic events experienced during childhood and/or adolescence—is causally associated with increased risk of psychotic experiences (PEs) in adulthood. ^1–5^

Individuals who have experienced DT are three times more likely to develop a psychotic disorder than those who have not. DT constitutes approximately one-third of the attributable risk fraction for psychosis.^1^^,^^3^ Adult survivors of DT are at increased risk of adverse prognostic outcomes, including more severe psychotic illness, poorer response to treatment, and increased morbidity and mortality.^6^ Despite this evidence, there is a lack of understanding of the mechanisms underlying this association.

Traumatic experiences are threatening to one’s survival, physical integrity, and sense of self. They engage the brain’s threat and memory systems, which aim to mitigate such threats.^7^ Longstanding evidence from human and animal studies demonstrate that the amygdala, hippocampus, and the prefrontal cortex are involved in learning and memory processes. These processes enable organisms to detect, learn, and respond to threats in a context-dependent manner.^8^^,^^9^ Research suggests that the perirhinal cortex processes information on specific items, the parahippocampal cortex processes information on the items in relation to their context. Information from these regions are then bound into a single episodic memory representation by the hippocampus.^10^ The dual representation model (utilizing findings from neuroimaging studies) proposes that heightened stress causes upregulation of the amygdala, and downregulation of the hippocampus, reducing associative memory of the event and increasing the risk of subsequent trauma memory intrusions.^9^

Relatedly, brain structure and function underlying threat and memory processing are susceptible to the effects of DT.^11–13^ Neuroimaging studies have reported DT to be associated with reductions in hippocampal volumes,^12^ and alterations in amygdala volume, varying depending on the type and timing of trauma experienced.^11^^,^^12^^,^^14^ It has been hypothesized that DT produces a small enlargement of the amygdala, but also sensitizes to subsequent stressors that result in a graded reduction in volume.^12^ Multiple longitudinal studies report DT to be associated with an increase in amygdala volume at baseline, followed by a more reliable reduction in amygdala volumes associated with later trauma exposure.^15^ It has also been hypothesized that the association between DT and reduced hippocampal volume may be due to the reduced processing of the contextual elements of the threatening experience.^16^

In parallel, there is evidence from human neuroimaging studies implicating structural alterations of brain regions involved in threat and memory processing in psychosis. Meta-analyses have reported reductions in amygdala, hippocampal, and ventromedial prefrontal cortex (vmPFC) volumes in individuals with schizophrenia.^17^^,^^18^ Recent meta-analyses have indicated that distinct biological mechanisms relating to threat processing may underly the association between DT and psychosis.^19^ One of these meta-analyses reviewing studies of schizophrenia patients found an association between DT and reduced volume in the prefrontal cortex. While they did not find any association between DT and reduced volume in the amygdala or hippocampus, they did find DT to be associated with altered activity in these regions.^19^ A recent meta-analysis reported DT to be associated with reduced right hippocampal volume and prefrontal cortex volume in youth at clinically high risk of psychosis.^15^ They also reported that former longitudinal studies found increased amygdala volume at baseline post DT, but decreased volume during adulthood.^15^

Cognitive theories of psychosis highlight the roles of altered threat and memory processing in underpinning psychotic symptoms. For example, altered attentional processing of threat-related stimuli leads to threatening interpretations in response to anomalous experiences, contributing to the development of paranoid delusions.^20^ Impairments in episodic memory processing at the time of trauma may result in intrusions from stored traumatic memories lacking contextual cues that give rise to hallucinations and delusions.^21^

Taken together, we hypothesized that DT exposure results in alterations in brain structures involved in threat and memory processing, potentially increasing the risk of future psychosis. To test this hypothesis in a well-characterized birth cohort, we investigated the effect of DT (assessed prospectively) on volumes of brain structures involved in threat and memory processing—the amygdala, vmPFC, striatum, hippocampus, parahippocampal cortex, and perirhinal cortex. We then examined their potential role in the association between DT and PEs in adulthood.^21^ We hypothesized that (1) DT is associated with increased PEs in adulthood, (2) DT is associated with alterations in brain regions involved in threat and memory processing, and (3) alterations in these brain regions are associates with PEs. As exploratory analyses, we also assessed the associations between the number of trauma types experienced, and the timing of DT with brain regions involved in threat and memory processing.

## Methods

### Sample

All participants were part of the Avon Longitudinal Study of Parents and Children (ALSPAC; http://www.bristol.ac.uk/alspac/), a pregnancy and birth cohort identifying factors influencing developmental outcomes.

Consistent with another study,^22^ MRI data were available and combined for 434 participants from two ALSPAC imaging studies. One of these imaging studies was based on PEs^23^ (*n* = 252), and the other ALSPAC imaging study recruited on the basis of polygenic risk scores ^24^(*n* = 196), with 14 participants taking part in both of these studies. Of the total 434 independent participants with MRI data from these studies, 419 participants with complete data from questionnaires relating to traumatic experiences collected at 0-17 years (see below), and PEs (see below) were included in the study.^23^^,^^25^ Participants provided written informed consent for the use of data collected from the study, following the recommendations of the ALSPAC Ethics and Law Committee at the time.

### Measures

#### Psychotic Experiences

PEs were assessed at age 18, using the semi-structured interview (PLIKSi), conducted by trained psychologists following the Schedules for Clinical Assessment in Neuropsychiatry guidelines.^26^ The PLIKSi comprises 12 core questions measuring the occurrence of hallucinations, delusions, and thought interference over the previous 6 months. Interviewers rated experiences as having “absent,” “suspected,” “definitely present,” and “clinically present” PE. Experiences mentioned had to have occurred at least once per month over the last 6 months. Individuals with “clinically present” PEs met operational criteria for clinical disorder if they had a diagnosis of psychosis, or was based on information relating to (1) social or educational decline, (2) frequency and distress of experiences, and (3) the effect of symptoms on functioning and help seeking from mental health services. Suspected, definitely present and clinical groups had PEs that were not attributable to the effects of sleep or fever. These 4 categories (no PEs > suspected PEs > definite PEs > psychotic disorder) were used for ordinal regressions, and were computed as a linear variable (from 0 to 4) for linear regressions.

#### Trauma Variables

Trauma variables were derived from assessments completed through self-report by participants or by their parents (eMethods in the Supplementary material). Data were collected at 3 separate time points (0-4.9 years, 5-10.9 years, and 11-17 years). Trauma types included emotional, physical and sexual abuse, emotional neglect, domestic violence, and bullying. There was no self-report assessment of emotional neglect between 0 and 4.9 years, so only data from 5-10.9 and 11-17 years were used. Three trauma variables were derived to represent (1) exposure to any trauma type between 0 and 17 years (derived as a binary variable), (2) the number of types of traumas experienced (with the number of trauma types experienced ranging from 0-6), and (3) when this trauma occurred; during childhood (0-10.9 years), adolescence (11-17 years) or both time periods (ie, 0 = no trauma, 1 = trauma exposure during childhood, 2 = trauma exposure during adolescence, 3 = exposure during both periods).

#### Confounding Variables

Data on sex, age at scanning, and total intracranial volume (TIV) were collected. We also controlled for a range of potential confounders of the trauma-psychosis association that have been reported as significant variables in a study of the complete ALSPAC sample.^2^ This included maternal educational status (measured as achieving less than O-levels, a secondary school-leaving qualification exam in the United Kingdom, achieving O-levels, and achieving higher than O-levels), household income (based on equivalized income reported between 33-47 months of age separated into quintiles), crowding index, and IQ at 8 years old. While analysis conducted with the primary confounders (age at scan, sex, TIV) used the original dataset, analysis including the additional confounders used an imputed dataset, consistent with analysis undertaken in a previous ALSPAC paper.^2^. Ten imputed datasets were created using the “mice” package, due to 25% missingness in these variables (maternal education status, household income, crowding index, and IQ at 8 years old). We remained conservative and did not impute for trauma, psychosis, or sMRI measures as they are our main measures relating to our key hypotheses.

#### MRI Acquisition, Preprocessing, and Volumetric Measures

All imaging data were acquired at the Cardiff University Brain Imaging Centre on a 3-T General Electric SIGNA HDx (GE Medical Systems, Milwaukee, WI, United States). Details of the acquisition parameters, sMRI preprocessing are provided in the eMethods in the Supplementary material. Those with PEs and controls from the same cohort without PEs were invited to undergo structural MRI (at one time) between age 19 and 24, with a mean age at scan of 21.2 (SD 1.45) years.

Brain volumes of interest included TIV as well as left and right amygdala, hippocampus, parahippocampal cortex, perirhinal cortex, vmPFC, and striatum (Figure 1). Total volumes for all structures were extracted using the MarsBar (v.0.44)^27^ toolbox on SPM, using region of interest (ROI) masks that were created from the AAL Atlas in WFU Pickatlas.^28^ The parahippocampal cortex and perirhinal cortex masks were extracted from open access data^29^ on Neurovault (https://identifiers.org/neurovault.collection:3731).

**Figure 1:**
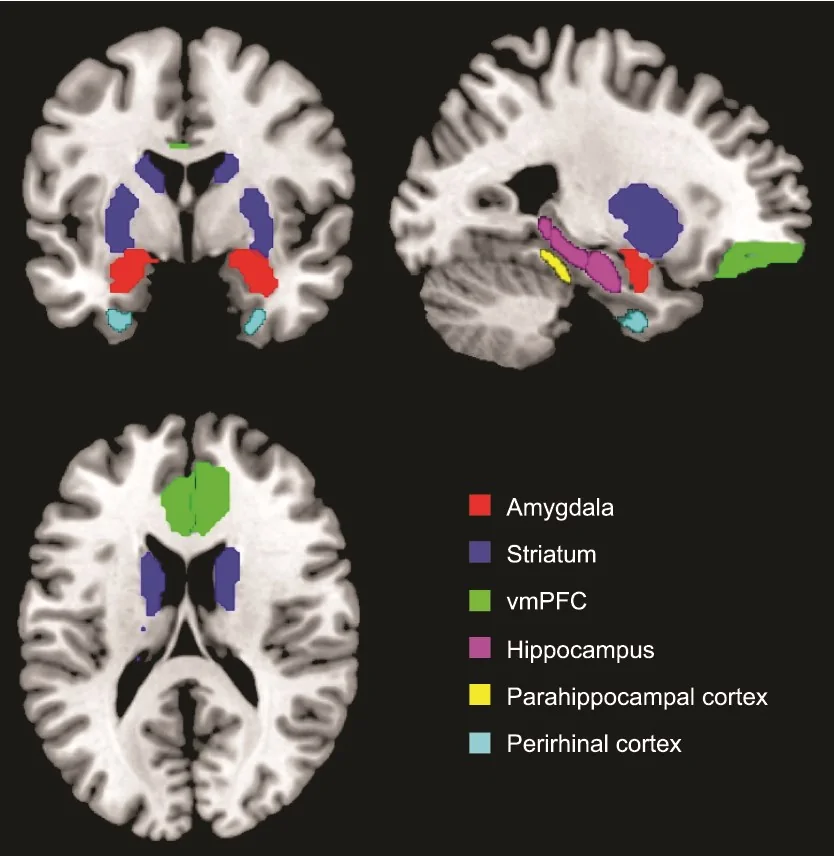
Threat and Memory Processing Region of Interests: Amygdala; Striatum; vmPFC; Hippocampus; Parahippocampal Cortex; Perirhinal Cortex

#### Statistical Analysis

Analyses were conducted using R (version 4.1.2). Ordinal logistic regression was used to calculate odds ratios (ORs) and 95% CIs for PEs (4-point ordinal scale: no PEs > suspected PEs > definite PEs > psychotic disorder) associated with exposure to DT (1) any trauma type experienced (Y/N), (2) number of trauma types experienced (0-6), (3) timing of trauma (none, childhood, adolescence, both) before and after adjusting for confounding factors.

We used hierarchical linear regression to examine the associations between exposure to DT and ROI volumes, and the association between ROI volumes and PEs, modeled as linear terms. Hierarchical regressions included confounders formerly mentioned. We also examined cumulative risk effect between number of trauma types experienced, modeled as linear terms, and timing of trauma, modeled as dummy variables, with ROI volumes. Bonferroni was used to correct for multiple comparisons in all analyses, and we report adjusted *P* values only.

## Results

### Study Sample

The sample of 419 participants included 248 (59.2%) females and 171 (40.3%) males with a mean age at scan of 21.2 (SD 1.45) years. Demographic data are presented in Table 1. As summarized in Table 1, 277 (66.1%) participants reported exposure to DT. A total of 152 (36.4%) participants were rated as having suspected (*n* = 47, 11.2%), definite (*n* = 71, 17.2%), or clinical (*n* = 34, 8.1%) levels of PEs at 18 years.

**Table 1:** Exposure to Trauma and Psychotic Experiences, by Type and Timing of Trauma

		**PLIKSi**					
	**All** **(*n* = 419)**	**None** **(*n* = 266)**	**Suspected** **(*n* = 47)**	**Definite** **(*n* = 71)**	**Clinical** **(*n* = 34)**	**OR (95% CI)**	***P*-value**
Age (mean, SD)	21.2(1.5)	21.5 (1.4)	20.8 (1.1)	20.9 (1.6)	20.1 (0.9)		<.001
Sex (% female)	59.2	56.6	66.0	56.3	76.5		.09
Maternal education	1.09 (.38)	1.09 (.36)	1.06 (.32)	1.11 (.43)	1.18 (.45)		.78
Household income	4.30 (2.4)	4.16 (2.3)	4.89 (2.6)	4.38 (2.7)	4.38 (2.4)		.34
Crowding index	2.32 (.88)	2.28 (.86)	2.40 (.77)	2.24 (.93)	2.74 (.96)		.06
IQ	111 (15.4)	112 (15.4)	108 (14.0)	111 (15.2)	109 (17.1)		.16
Total intracranial volume (mean, SD)	1449 (143)	1456 (138)	1433 (147)	1463 (147)	1391 (165)		.038
Trauma exposure (*n*, %)	277 (66.1)	163 (61)	40 (85.1)	48 (67.6)	26 (76.5)	1.64 (1.04-2.59)	.04
Physical abuse	112 (26.7)	61 (22.8)	13 (27.7)	24 (33.8)	14 (41.2)	1.77 (1.15-2.71)	.01
Emotional abuse	106 (25.3)	57 (21.3)	19 (40.4)	19 (26.8)	11 (32.4)	1.59 (1.03-2.43)	.03
Bullying	139 (33.2)	69 (25.8)	26 (55.3)	30 (42.3)	14 (41.2)	2.06 (1.38-3.09)	<01
Sexual abuse	56 (13.4)	26 (9.7)	7 (14.9)	13 (18.3)	10 (29.4)	2.25 (1.28-3.9)	.01
Domestic violence	91 (21.7)	51 (19.1)	10 (21.3)	21 (29.6)	9 (26.5)	1.53 (0.97-2.4)	.07
Emotional neglect	39 (9.3)	17 (6.4)	8 (17)	6 (8.5)	8 (23.5)	1.94 (0.98-3.78)	.05
Trauma type							
0	142 (33.9)	104 (39)	7 (14.9)	23 (32.4)	8 (23.5)		
1	1	82 (30.7)	16 (34)	19 (26.8)	8 (23.5)	0.85 (0.55-19)	0.45
2	83 (19.8)	53 (19.9)	13 (27.7)	9 (12.7)	8 (23.5)	0.95 (0.58-13)	0.84
≥3	69 (16.5)	28 (10.5)	11 (23.4)	20 (28.2)	10 (29.4)	2.9 (1.79-4)	<.001
Linear trend						0.16 (0.08-0.24)	<.001
Trauma timing							
None	142 (33.9)	104 (39)	7 (14.9)	23 (32.4)	8 (23.5)		
Childhood	110 (26.3)	77 (28.8)	12 (25.5)	13 (18.3)	8 (23.5)	1.06 (0.59-1.88)	.85
Adolescence	52 (12.4)	30 (11.2)	10 (21.3)	8 (11.3)	4 (11.8)	1.39 (0.71-2.75)	.34
Both	115 (27.4)	56 (21)	18 (38.3)	27 (38)	14 (41.2)	2.57 (1.51-4.37)	<01
Linear trend						0.13 (0.05-0.21)	<.001

All *p* values are adjusted post Bonferroni correction.

### DT Exposure and Psychotic Experiences

Exposure to any type of DT was associated with increased odds of PEs (OR = 1.64; 95% CI, 1.04-2.59, *P* = .035) (Table 1). There was evidence supporting cumulative risk effects, as the number of types of DT experienced was associated with increased PEs (*B* = 0.160, 95% CI, 0.084-0.237, *P* < .001), and experiencing trauma during both childhood and adolescence were associated with increased PEs (OR = 2.57, 95% CI, 1.51-4.37, *P* < .001). Experiencing trauma during only childhood (OR = 1.06, 95% CI, 0.594-1.88, *P* = .85) or adolescence (OR = 1.39, 95% CI, 0.707-2.75, *P* = .34) was not associated with increased PEs.

### DT Exposure and Brain Structures Involved in Threat and Memory Processing

Exposure to DT was associated with reduced left amygdala volume in adulthood (*B* = −0.011, *P* = .02, Table 2 and Table S1), with evidence supporting a cumulative risk effect, whereby increased number of trauma types experienced was associated with greater reductions in left amygdala volumes (*B* = −0.006, *P* = .01). These associations were not seen in right amygdala, vmPFC, or striatal volumes (all *P*s > .05). Exposure to DT was not associated with brain regions involved in memory processing (all *P*s > .05).

**Table 2:** Association Between DT and Brain Structures Involved in Threat and Memory Processing

**ROI**	**B**	***P*-value**
Amygdala		
Both	−0.007	.08
Left	−0.011	.02
Right	−0.003	.44
vmPFC		
Both	0.001	.81
Left	0.0 01	.74
Right	0.000	.92
Striatum		
All	0.005	.59
Associative	0.002	.71
Limbic	0.001	.78
Sensorimotor	0.007	.20
Hippocampus		
Both	−0.005	.08
Left	−0.005	.08
Right	−0.004	.12
Parahippocampal cortex		
Both	−0.005	.06
Left	−0.004	.08
Right	−0.005	.07
Perirhinal cortex		
Both	−0.006	.31
Left	−0.006	.34
Right	−0.005	.40

All *p* values are adjusted post Bonferroni correction.

### Brain Structures Involved in Threat and Memory Processing and Psychotic Experiences

Reduced bilateral amygdala volumes were associated with an increased odds of PEs, driven by the left amygdala (OR = 0.001, 95% CI, 0.000-0.154, *P* = .006) (Table 3). Reduced vmPFC volumes were associated with significant but non-substantial increases in odds of PEs (all ORs < .0001, all *P*s < .01). We did not observe any significant associations between the striatum, nor regions involved in memory processing, with PEs.

**Table 3:** Association Between ROI Volumes and Psychotic Experiences

**ROI**	**OR**	**95 % CI lower**	**95 %CI upper**	***P*-value**
Amygdala				
Both	0.000	8.80E-07	0.118	.008
Left	0.001	1.43E-05	0.154	.006
Right	0.003	6.67E-06	0.961	.050
PFC				
Both	0.000	4.36E-09	0.025	.004
Left	0.000	2.32E-08	0.055	.007
Right	0.000	8.33E-09	0.033	.005
Striatum				
All	0.379	3.94E-02	3.644	.406
Associative	0.123	1.19E-03	12.696	.380
Limbic	0.032	1.95E-04	5.285	.192
Sensorimotor	7.835	1.29E-01	474.870	.330
Hippocampus				
Both	0.083	2.39E-05	289.337	.551
Left	0.166	1.12E-04	246.559	.630
Right	0.069	2.12E-05	222.790	.518
Parahippocampal cortex				
Both	0.004	8.72E-07	20.666	.212
Left	0.048	1.08E-05	210.816	.481
Right	0.003	1.29E-06	4.943	.125
Perirhinal cortex				
Both	0.046	8.78E-04	2.415	.132
Left	0.198	7.63E-03	5.158	.334
Right	0.028	5.52E-04	1.373	.076

All *p* values are adjusted post Bonferroni correction.

## Discussion

In this study, we examined the association between DT on brain structures involved in threat and memory processing in a large, well-characterized birth cohort. We also examined their potential role in the association between DT and PEs in adulthood. DT measured longitudinally and controlling for a range of confounders was associated with increased PEs in adulthood. DT was also associated with reduced left amygdala volume, and reduced bilateral amygdala volume (driven by the left amygdala) was associated with increased PEs. Taken together, our findings potentially suggest a threat-based neurobiological mechanism that may underly the association between DT and PEs.

As previously reported in the complete ALSPAC sample,^2^ DT was associated with increased PEs in adulthood. DT was also associated with reduced left amygdala volumes, with evidence supporting a cumulative risk effect. Structural alterations in this brain region is thought to be an experience-dependent modification in response to trauma,^12^ arising from an interplay between excitatory and inhibitory circuits following sensory experiences during sensitive periods of brain development.^30^ The amygdala plays a central role in threat detection and response,^8^ processing emotional information^31^ and studies have found an inverse association between amygdala volume and activation during threat detection.^32^ Considering this, our findings provide some evidence supporting the hypothesis that there is an association between DT and alterations in brain structures involved in threat and processing.

There were no significant associations between DT and brain regions associated with memory processing, nor these regions with PEs. This is not consistent with some neuroimaging studies reporting reduced hippocampal and parahippocampal volume in people exposed to DT.^15^^,^^33^ This is also inconsistent with studies reporting reductions in hippocampal volume to predate conversion to psychosis,^34^ or to reduce later on in psychosis development.^17^^,^^18^^,^^20^^,^^21^^,^^35–37^ However, one meta-analysis reported that no study (using a whole-brain or ROI approach), found an association between hippocampus volume and childhood trauma in people with schizophrenia. They stated that this non-significant finding may have been due to confounding variables not being controlled for, including urbanicity and substance abuse (eg, cannabis). Both of these variables were not controlled for in this current study. Therefore, future studies could more directly consider how other confounding factors may play a role in the association between DT, hippocampal volume, and PEs.

Compared to the hippocampal volume findings, the non-significant results found in the perirhinal cortex are more consistent with former studies. The lack of significant results in the perirhinal cortex could suggest that trauma-induced brain changes may be more specific to regions associated with the contextual elements of memory processing, supporting models suggesting that traumatic memories are not bound by spatial or temporal context.^16^ Additionally, only a few studies have reported reduction in perirhinal volume in psychosis samples.^38^

### Implications of Findings

This study provides evidence to support threat processing as a potential biological mechanism underpinning the association between DT and PEs. Given that threat processing can be indexed and measured via neurocognitive tasks, these may act as potential prognostic markers that can be used to guide personalized therapeutic strategies. Indeed, these findings support existing psychological and pharmacological interventions that target threat processing, which are trauma-focused.^39–41^

### Strengths and Limitations

This study has many strengths, including its use of a large prospective population-based birth cohort, with multiple measures of trauma at different stages of childhood and adolescence to minimize recall bias. We also controlled for a range of confounders of the DT-psychosis association. The use of semi-structured interviews (compared to self-report questionnaires) to assess PEs increased the validity of the outcome, as well as confidence in the general inferences made.

Some limitations in this study should be acknowledged. First, the collection of neuroimaging data after DT and psychosis measures precludes us from making inferences about the causal relationships between DT, alterations in brain structures and subsequent PEs. Longitudinal studies with repeated measures of both imaging and PEs are needed to determine whether alterations in brain structure identified predict the onset of PEs in later life. Second, this study did not account for the effect of gene–environment correlations, where, for instance, genes that contribute to psychosis risk may also increase the likelihood of experiencing DT, warranting the need for genetically informed studies. Third, given that this study recruited a subsample of individuals from a birth cohort, where, as with most cohort studies, there was attrition over time, the study is susceptible to the possible effects of selection bias. Considering there was no self-report assessment of emotional neglect between 0 and 4.9 years and that trauma data from this subgroup were reported by parents, it is possible that the prevalence of abuse in the youngest cohort were not completely captured. While VBM has been widely used to measure anatomical volume, FreeSurfer segmentation could have been a more robust method of providing direct volumetric measurements of the regions of interest analyzed. Our analyses used VBM, which differs from FreeSurfer in its approach to measure grey matter volume. VBM estimates voxel-wise grey matter concentrations by spatially normalizing brain images to a template and, when modulation is applied, scales voxel values by the degree of local expansion or contraction to preserve regional volume. FreeSurfer, in contrast, provides direct volumetric estimates in native space using explicit anatomical boundaries. These methodological differences mean mild discrepancies may arise between our study results compared to studies using Freesurfer. However, studies have shown that ROI-based estimates of hippocampal and amygdala volume generated with VBM are comparable to those produced by FreeSurfer when the same ROI definitions are used.^42^ While we only included confounders that were found to be significant in a previous ALSPAC paper measuring the association between trauma and PEs^2^ another variable that could be included as a confounder in future research is family history of psychosis. Additionally, the methods used (eg, hierarchical regression) may not have fully utilized the longitudinal nature of the data. Future research should consider mixed-effects or growth models to better capture within-subject changes over time. Lastly, it would have been beneficial to measure PTSD within the cohort to delineate the relationship between DT and psychosis.

## Conclusion

The findings in this study provides support of a causal association between DT and altered structure of brain regions involved in threat processing. Our findings also suggest a potential neurobiological mechanism underlying the association between DT and PEs in adulthood. Future work should use longitudinal neuroimaging data to examine temporal associations between DT, altered threat and memory processing and PEs. It could also include behavioral and functional measures of threat and memory processing to strengthen the mechanisms raised.

## Supplementary Material

alspac_dt_roi_psychosis_supplement_v2_sbaf184
