# Supplementary material for: The Effect of Developmental Trauma on Brain Structures Involved in Threat and Memory Processing and Its Relation to Psychotic Experiences in Adulthood
Source: Schizophr Bull. 2025 Oct 28;52(5):sbaf184. doi: 10.1093/schbul/sbaf184 (PMC13645828; doi:10.1093/schbul/sbaf184)
Supplement: Supplementary file 1 [file alspac_dt_roi_psychosis_supplement_v2_sbaf184.docx]

**Supplementary Online Content**

**eMethods**

**eTable 1.** Association between DT and brain structures involved in threat and memory processing

**eMethods**

*Participants*

All pregnant women with an expected delivery from 1^st^ April 1991 to 31^st^ December 1992 (approximately 12,000) who resided in the county of Avon, Southwest of England were invited to participate. Participants have undergone multiple assessments with extensive data collected on biological, social and psychological (including psychopathological). ^49^ Informed consent was obtained before imaging, with ethical approval provided by the Cardiff University School of Psychology Ethics Committee and the ALSPAC Ethics and Law Committee.

*Assessment of psychotic experiences (PEs)*

The semi-structured interview (PLIKSi) consists of 12 core questions that assesses delusions (grandiose ability, control, reference, thoughts being read, being spied on, persecution and unspecified delusions), auditory hallucinations, visual hallucinations and thought inference (thought withdrawal, broadcasting, and insertion). Trained psychologists conducted the interviews and rated responses following the Schedules for Clinical Assessment in Neuropsychiatry guidelines, using probing and cross-questioning to establish the absence or presence of psychotic experiences. Interviewers rated experiences as absent, suspected or definitely present. Individuals with psychotic experiences that were rated as ‘definitely present’, had these experiences at least once per month over the last 6 months. A subset of those with definitely present PEs met operational criteria for ‘clinical disorder’ based on evidence of social or educational decline, frequency and distress of experiences, and their effect on functioning, help seeking from mental health services, or a diagnosis of psychosis. Suspected, definitely present and clinical groups had psychotic experiences that were not attributable to the effects of sleep or fever.

*Brain imaging*

All imaging data were acquired at the Cardiff University Brain Imaging Centre (CUBRIC) on a 3-T General Electric SIGNA HDx (GE Medical Systems, Milwaukee, WI, USA) using an 8-channel head coil for radiofrequency reception. Gray matter volume was derived from high-resolution, 3D fast-spoiled gradient-echo T1-weighted isotropic images acquired with oriented to the AC–PC line (TR = 7.8 ms, TE = 3 ms, inversion time = 450 ms, flip angle = 20°, field of view= 256 mm × 256 mm x 192 mm, 1 mm isotropic resolution).

Structural magnetic resonance imaging (sMRI) preprocessing was conducting with spatial smoothing using a Gaussian kernel of 8 mm full width at half maximum using SPM 12 standard routines (SPM version 12; [www.fil](http://www.fil).ion.ucl.ac.uk/spm). T1-weighted images were segmented using default tissue probability maps of white and gray matter, and segmentation parameters were then imported into DARTEL ^50^ producing rigidly aligned gray matter images. A mean image template of all participants was reiteratively created and normalised to the template defined by the Montreal Neurological Institute (MNI). Resulting deformations were applied to transform the segmented gray matter images and smoothed with a Gaussian filter of 8-mm full-width half-maximum.

**eTable 1.** Association between developmental trauma and brain structures involved in threat and memory processing

|  | **Trauma exposure** | | **Trauma types** | | **Trauma timing** | | | | | |
| --- | --- | --- | --- | --- | --- | --- | --- | --- | --- | --- |
|  |  |  |  |  | **Childhood** | | **Adolescence** | | **Both** | |
| **ROI** | **B** | **P Value** | **B** | **P Value** | **B** | **P Value** | **B** | **P Value** | **B** | **P Value** |
| **Amygdala** |  |  |  |  |  |  |  |  |  |  |
| Both | -0.007 | 0.08 | -0.001 | 0.37 | -0.008 | 0.09 | -0.014 | 0.01 | -0.002 | 0.72 |
| Left | -0.011 | 0.02 | -0.003 | 0.07 | -0.010 | 0.07 | -0.015 | 0.04 | -0.009 | 0.10 |
| Right | -0.003 | 0.44 | 0.001 | 0.72 | -0.005 | 0.22 | -0.014 | 0.01 | 0.005 | 0.25 |
| **vmPFC** |  |  |  |  |  |  |  |  |  |  |
| Both | 0.001 | 0.81 | 0.001 | 0.34 | 0.000 | 0.90 | -0.005 | 0.29 | 0.003 | 0.32 |
| Left | 0.001 | 0.74 | 0.001 | 0.36 | 0.000 | 0.96 | -0.006 | 0.20 | 0.005 | 0.17 |
| Right | 0.000 | 0.92 | 0.001 | 0.36 | 0.001 | 0.85 | -0.004 | 0.43 | 0.002 | 0.62 |
| **Striatum** |  |  |  |  |  |  |  |  |  |  |
| All | 0.005 | 0.59 | 0.004 | 0.24 | 0.004 | 0.75 | -0.020 | 0.18 | 0.018 | 0.11 |
| Associative | 0.002 | 0.71 | 0.001 | 0.71 | 0.003 | 0.60 | -0.007 | 0.36 | 0.004 | 0.44 |
| Limbic | 0.001 | 0.78 | 0.002 | 0.18 | -0.001 | 0.80 | -0.012 | 0.06 | 0.010 | 0.05 |
| Sensorimotor | 0.007 | 0.20 | 0.004 | 0.03 | 0.006 | 0.34 | -0.003 | 0.69 | 0.012 | 0.06 |
| **Hippocampus** |  |  |  |  |  |  |  |  |  |  |
| Both | -0.005 | 0.08 | -0.001 | 0.25 | -0.008 | 0.01 | -0.003 | 0.48 | -0.002 | 0.56 |
| Left | -0.005 | 0.08 | -0.002 | 0.09 | -0.008 | 0.04 | -0.003 | 0.57 | -0.004 | 0.29 |
| Right | -0.004 | 0.12 | 0.000 | 0.67 | -0.009 | 0.01 | -0.003 | 0.43 | 0.000 | 1.00 |
| **Parahippocampal cortex** | |  |  |  |  |  |  |  |  |  |
| Both | -0.005 | 0.06 | -0.001 | 0.27 | -0.007 | 0.03 | -0.004 | 0.34 | -0.003 | 0.27 |
| Left | -0.004 | 0.08 | -0.001 | 0.25 | -0.006 | 0.07 | -0.002 | 0.64 | -0.004 | 0.17 |
| Right | -0.005 | 0.07 | -0.001 | 0.35 | -0.007 | 0.03 | -0.005 | 0.22 | -0.003 | 0.46 |
| **Perirhinal cortex** |  |  |  |  |  |  |  |  |  |  |
| Both | -0.006 | 0.31 | -0.002 | 0.25 | -0.004 | 0.61 | -0.004 | 0.64 | -0.009 | 0.21 |
| Left | -0.006 | 0.34 | -0.003 | 0.19 | -0.004 | 0.63 | 0.001 | 0.96 | -0.012 | 0.13 |
| Right | -0.005 | 0.40 | -0.001 | 0.50 | -0.003 | 0.67 | -0.009 | 0.32 | -0.005 | 0.51 |
